# Identification of Potential Hub Genes and miRNA-mRNA Pairs Related to the Progression and Prognosis of Cervical Cancer Through Integrated Bioinformatics Analysis

**DOI:** 10.3389/fgene.2021.775006

**Published:** 2021-12-22

**Authors:** Mingxu Fu, Yongyan Pei, Fang Lu, Huici Jiang, Yingying Bi, Jiajing Cheng, Jinlong Qin

**Affiliations:** ^1^ Department of Obstetrics and Gynecology, Shanghai Fourth People ’s Hospital, School of Medicine, Tongji University, Shanghai, China; ^2^ School of Medicine and Chemical Engineering, Guangdong Pharmaceutical University, Guangzhou, China

**Keywords:** cervical cancer, miR-197-3p/TYMS, proliferation, apoptosis, invasion and migration

## Abstract

In recent years, the incidence and mortality of cervical cancer have increased worldwide. At the same time, increasing data have confirmed that miRNA-mRNA plays a positive or negative regulatory role in many cancers. This study attempted to screen effective miRNA-mRNA in the progression of cervical cancer, and to study the mechanism of miRNA-mRNA in the progression of cervical cancer. The expression profile data of GSE7410, GSE 63514, GSE 86100 and TCGA-CESC were downloaded, and 34 overlapping differentially expressed genes (22 up-regulated and 12 down-regulated) and 166 miRNAs (74 down-regulated and 92 up-regulated) were screened through limma package. Then, miR-197-3p/TYMS pairs were obtained by PPI, functional enrichment, Kaplan-Meier plotter analysis, Cox univariate and multivariate analysis, risk modeling, WGCNA, qPCR and dual-luciferase experiments. The results showed that TYMS was an independent prognostic factor of cervical cancer, and its expression level was negatively correlated with cervical cancer tissue grade (TMN), tumor grade, age, microsatellite stability and tumor mutation load, and positively correlated with methyl expression in DNMT1, DNMT2, DNMT3A and DNMT3B. Functional experiments showed that TYMS knockout could promote the proliferation, migration and invasion of HeLa cells and reduce apoptosis. Overexpression of TYMS showed the opposite trend, miR-197-3p was negatively correlated with the expression of TYMS. MiR-197-3p inhibitor reversed the effect of si-TYMS on the proliferation of HeLa cells. In conclusion, these results reveal that TYMS plays a very important role in the prognosis and progression of cervical cancer, and has the potential to be thought of as cervical cancer biomarkers. At the same time, miR-197-3p/TYMS axis can regulate the deterioration of cervical cancer cells, which lays a foundation for the molecular diagnosis and treatment of cervical cancer.

## Introduction

Cervical cancer is the most common female malignancy worldwide, and directly causes high incidence and mortality rates in women (Wang et al.,2020). According to the database of the International Agency for Research on Cancer, there were more than 500,000 new cases of cervical cancer and 311,000 deaths in 2018 (Stelzle et al.,2021). To date, surgery and radiotherapy are still the main treatment methods for cervical cancer, but recurrence, metastasis and drug resistance often occur after treatment (Sharma et al.,2020; Shin et al.,2020). The mechanism of cervical cancer is complex. Different genes, RNAs and signaling pathways are related to the tumorigenicity of cervical cancer (Huang et al., 2020; Rasmi and Sakthivel, 2020). Therefore, it is worthwhile to find new methods to study the basic mechanism of cervical cancer to improve treatment.

RNA plays an important role in the regulation of several major biological processes affecting tumorigenesis and progression, and has always been at the forefront of tumor molecular mechanism research (Goodall and Wickramasinghe, 2021; Barbieri & Kouzarides, 2020). The combination of high-throughput technology and bioinformatics analysis can provide researchers with valuable data available in the form of public datasets to search for biomarkers and therapeutic targets (Liu et al.,2020; Chen et al., 2008). Gene Expression Omnibus (GEO) facilitates the submission, storage, and retrieval of heterogeneous datasets from high-throughput gene expression and genomic experiments (Clough and Barrett, 2016; Yang et al., 2020). The Cancer Genome Atlas (TCGA) is a comprehensive dataset that provides a unified data analysis pipeline for further exploration of oncogene signaling changes and their associated significance in cancer patient outcomes (Hutter and Zenklusen, 2018; Linehan and Ricketts, 2019). Therefore, the combination of GEO and TCGA data sets may provide an important perspective for the study of new biomarkers. There have been many reports on screening tumor biomarkers based on GEO and TCGA data, and a series of markers with high specificity and sensitivity have been found (Ren et al., 2020; Xu et al.,2020). Compared with traditional screening methods, bioinformation-based analysis of high-throughput data enables researchers to obtain stable and reliable biomarkers in a large number of clinical samples.

In this study, we combined cervical cancer in GEO database and TCGA databases to screen differentially expressed genes and differentially expressed miRNAs. Based on the differentially expressed genes, the candidate gene TYMS was screened by functional annotation, pathway analysis, protein interaction (PPI) network construction, prognosis analysis, risk assessment model and WGCNA. Then, potential candidate pairs (miR-197-3p/TYMS) were screened by dual-luciferase experiment and qPCR. Subsequently, the effects of TYMS and miR-197-3p/TYMS on the progression of cervical cancer were analyzed by gain-of-function analysis. We hope to provide more extensive data support for the clinical application of TYMS and the miR-197-3p/TYMS axis in cervical cancer.

## Materials and Methods

### Data Collection and Preprocessing

The original datasets were downloaded from geo database to compare the mRNA expression and miRNA expression between cervical cancer and normal tissues. The expression profile data of GSE7410, GSE63514, GSE86100 and GSE9750 were downloaded based on different platforms (Table 1). The mRNA expression data of cervical cancer were downloaded from TCGA database for preprocessing. Clinical samples related to cervical cancer were selected. The data set includes 307 cervical cancer samples, 307 normal samples and corresponding clinical data. Then, Sangerbox tool 2.0 (http://sangerbox.com/Index) corrects and normalizes the original expression data background of each GEO dataset, and eliminates batch effects and other irrelevant variables.

**TABLE 1:** Information of GEO datasets.

Dataset	Platform		Tumor	Normal	References
GSE7410	GPL1708	Agilent-012391 Whole Human Genome Oligo Microarray G4112A	40	5	Biewenga et al. (2008)
GSE63514	GPL570	Affymetrix Human Genome U133 Plus 2.0 Array	28	24	den Boon et al. (2015)
GSE86100	GPL19730	Agilent-046064 Unrestricted Human miRNA V19.0 Microarray	8	4	Gao et al.2016
GSE9750	GPL96	Affymetrix Human Genome U133A Array	43	23	Scotto et al., 2008

### Differentially Expressed mRNAs and miRNAs Screening

The differentially expressed mRNAs and miRNAs between cervical cancer and normal tissues were identified by R software limma software package. The GEO dataset filtering criteria are: log2|Fold Change|>1, adjusted *p*-value< 0.05 and false discovery rate (FDR) < 0.05. The screening criteria of the TCGA dataset were: log2|Fold Change|>2, adjusted *p*-value< 0.05 and FDR<0.05. The ggplot2 and pheatmap packages were used to draw differential mRNAs and miRNAs volcano maps and heatmaps respectively. Then, a Venn diagram was used to find overlapping differentially expressed mRNAs.

### Gene Ontology and Kyoto Encyclopedia of Genes and Genomes Pathway Enrichment Analyses

To further analyze differentially expressed mRNAs, GO and KEGG enrichment analyses were performed using the DAVID online tool 6.8 (https://david.ncifcrf.gov/home.jsp) and Metascape (http://metascape.org/gp/index.html). GO enrichment analysis mainly annotates the biological process (BP), cytological component (CC) and molecular function (MF) of genes. KEGG enrichment analysis mainly predicts the signal pathways that may be involved. *p* < 0.05 was considered statistically significant.

### PPI and Key Module Selection

PPI network interactions were identified and constructed using the STRING online database 11.5 (http://string-db.org). An interaction confidence≥0.4 was considered significant. Then, the data were imported into Cytoscape software 3.8.0 for reprocessing. Subsequently, the molecular complex detection (MCODE) plug-in was used to filter hub modules in the PPI network. Then, GO and KEGG analyses of mRNAs in hub modules were performed using Metascape (http://metascape.org/gp/index.html).

### Prognostic Analysis

The clinical characteristic data of cervical cancer patients from TCGA were downloaded. First, we subtyped the downloaded cervical cancer samples, and then analyzed the survival of each subtype and each pathological parameter. Then, univariate Cox proportional hazards regression analysis was used to identify potential genes highly related to overall survival and to screen mRNAs related to the prognosis of cervical cancer (*p* < 0.05). Then, the data were further screened by multivariate Cox proportional hazards regression. A risk assessment model was constructed with key prognostic mRNAs as dependent variables to evaluate the clinical value of key prognostic mRNAs in predicting patient survival. The risk assessment model formula is: risk score = expression of mRNA1 × β_mRNA1_ + expression of mRNA2 × β_mRNA2_ +……+ expression of mRNAn × β_mRNAn_. *β* represents the multivariate Cox regression coefficient, which is obtained from the multivariable Cox proportional hazards regression model of each gene. According to the median risk score, patients with cervical cancer were divided into high and low-risk groups. Then the survival rates of the high and low-risk groups were analyzed. At the same time, a receiver operating characteristic (ROC) curve was constructed.

### Weighted Gene Coexpression Network Analysis

First, we preprocessed the GSE9750 data, removed the samples with incomplete clinical data, removed the outliers, and performed WGCNA in the Sangerbox tool 2.0 (http://sangerbox.com/Index). The soft threshold was set as *β* = 1, and a scale-free network was constructed. Then, we transformed the adjacency matrix into a topological overlap matrix (TOM). Next, we performed hierarchical clustering to identify modules, and each module contained at least 20 genes (min module size = 20). Subsequently, characteristic genes were calculated, hierarchical clustering modules were used, and similar modules were merged (abline = 0.25). Finally, we calculated the correlation between the module and clinical data to determine meaningful clinical modules.

### Gene Set Enrichment Analysis

We performed GSEA using normalized RNA SEQ data obtained by TCGA. GO and KEGG analyses of candidate genes were performed with Sangerbox tool 2.0 (http://sangerbox.com/Index) to study the possible biological functions of candidate genes. Adjusted *p* value < 0.05 and FDR <0.05 were considered to be statistically significant.

### Cell Culture and Transfection

HeLa cells were purchased from Beijing Beina Chuang lian Biotechnology Research Institute (Beijing, China). The cells were cultured in Dulbecco’s modified Eagle’s medium (DMEM, Invitrogen, United States). At the same time, 10% fetal bovine serum (Invitrogen, United States), 100 U/ml penicillin (Sigma, United States) and 100 g/ml streptomycin (Sigma, United States) were added. The cells were cultured in 5% CO_2_ at 37 C in a constant-temperature incubator. The eighth passage HeLa cells were used in this study. According to the instructions for HeLa cells, Lipofectamine 3,000 reagent (Invitrogen, United States) was used to transfect 50 nm siRNA (si-TYMS; RiboBio, China), pCDH-TYMS (RiboBio, China), 50 nm miR-197-3p mimic (mir100000 227-one to five, RiboBio, China), 100 nm miR-197-3p inhibitor (mir200000 227-one to five, RiboBio, China) and their NC control for 24 h.

### RNA Extraction and Real-Time Quantitative PCR

A total of 30 cervical cancer tissues and 30 adjacent normal tissues were collected (Supplementary Material S1). This study was approved by the hospital ethics committee and was carried out in accordance with the Declaration of Helsinki. In addition, each patient provided written informed consent. The cervical cancer tissue was cut off during surgery and immediately stored at 80°C until use.

Total RNA was extracted from tissues and cells using RNeasy Mini Kit (Qiagen, Germany). Then, the concentration of extracted RNA was determined with Nanodrop 2000 (Invitrogen, United States). The same amount of RNA was reverse transcribed into cDNA with Goldstar™ RT6 cDNA synthesis kit ver2 (TsingKe, China). Master qPCR mix (SYBR Green 1) (Beyotime, China) was used for qPCR detection. The expression of GAPDH was used as the endogenous control. Primer 5.0 software was used to design primers 5.0 (TYMS forward primer 5'-3': ACT​TGT​GCA​GAT​TAT​TCA​GGA​C, TYMS reverse primer 5'-3': ATT​CTT​CTG​TCG​TCA​GGG​T; GAPDH forward primer 5'-3':CAT​TTC​CTG​GTA​TGA​CAA​CGA, GAPDH reverse primer 5'-3': GGG​TCT​TAC​TCC​TTG​GAG​G). The primer sequences were sent to Sangon (China) for synthesis. For miRNA detection, U6 was used as the endogenous control, and the primers were purchased from RiboBiology (miR-197-3p: mqps0000765-1-100, RiboBio, China). QPCR detection was performed using bulge loop miRNA QRT PCR Starter Kit (RiboBio, China). MiRNA and mRNA expression levels were calculated by 2^−ΔΔCt^.qRT-PCR data were analyzed by GraphPad Prism, and each reaction was repeated 3 times. A *t* test was used in both groups (*p* < 0.05), and one-way ANOVA was used in more than two groups (*p* < 0.05).

### CCK8 Assays

Logarithmic growth HeLa cells were inoculated into 96-well plates at 4,000 cells/well per well. 24 h after transfection, CCK-8 reagent (10 μl per well, Beyotime, China) was added to all cells, and the cells were incubated for 3 h. Then, a multi-function microplate (Thermo, United States) was used to measure the absorbance at 450 nm. Each experiment was conducted three times.

### Wound-Healing Assay

HeLa cells at the logarithmic growth stage were inoculated into 96-well plates at 1×10^5^ cells per well. 24 h after transfection, the fused cell monolayer was scratched with 200 μl sterile pipette tip and then added to serum-free DMEM. The scratch was recorded by microscopy at 0 and 24 h, and the scratch closure rate was evaluated by ImageJ software.

### Transwell Assay

HeLa cells transfected for 24 h were collected for single-cell suspension, and 1×10^3^ cells were added to each upper chamber (Corning, United States) containing matrix gel. DMEM with 20% fetal bovine serum was added to lower chamber. After 24 h, the cells were fixed with 4% paraformaldehyde (Beyotime, China) and stained with 1% crystal violet (Beyotime, China). Cells were observed and counted under a light microscope.

### Cell Apoptosis

24 h after transfection, the HeLa cells were digested with trypsin to prepare single cell suspension. Then, the cells were gently washed with precooled PBS 3 times, and the cell concentration was maintained at 3 × 10^5^ cell/ml. The cells were then treated with annexin V-FITC & PI apoptosis detection kit (Sangon, China). After treatment, the number of apoptotic cells analyzed by Beckman flow cytometry (Beckman, United States).

### Luciferase Reporter Assay

HeLa cells were inoculated into 96-well plates and cotransfected with 100 ng of the dual-luciferase reporter constructs pmiR-RB-Report-TYMS 3'UTR (RiboBio, China) and miR-197-3p mimic (RiboBio, China). After incubation for 48 h, the supernatants of the cells were collected and the fluorescence value was determined using dual-luciferase reporter kit (Promega, United States).

### Western Blotting

48 h after the transfection of HeLa cells, the culture medium was removed. After PBS rinsing, the cells were lysed with protein lysate (Beyotime, China), and the total proteins were collected. Then the protein concentration was detected by BCA (Beyotime, China) protein quantitative kit. After adding proper amount of SDS loading buffer (Beyotime, China), denaturation was carried out in boiling water at 100°C for 5 min. Then 12% SDS-PAGE electrophoresis (Beyotime, China) was performed. Protein bands were transferred to PVDF membranes (Beyotime, China) by western transmembrane system. Subsequently, the PVDF membrane was sealed in 5% skim milk powder (Beyotime, China) and incubated for 4 h. After washing with TBST (Beyotime, China), the PVDF membrane was incubated with primary antibody at 4°C overnight. After washing, the membrane was incubated with the secondary antibody at room temperature for 60 min. Finally, the PVDF membrane was stained with western TMB substrate (Beyotime, China; P0211) and scanned. The antibodies used were β-actin (Beyotime, China; AF5003), TYMS (Abcam, United States, ab108995) and horseradish peroxidase labeled goat anti-rabbit IgG (Beyotime, China; A0208).

### Statistical Analysis

All data were processed by SPSS 25 and GraphPad Prism 8. The measurement data are expressed as mean ± standard deviation (SD). A *t* test and one-way ANOVA were performed between two groups and multiple groups, respectively. *p* < 0.05 was considered statistically significant.

## Results

### Identification of Differentially Expressed miRNAs and mRNAs in Cervical Cancer

**FIGURE 1:**
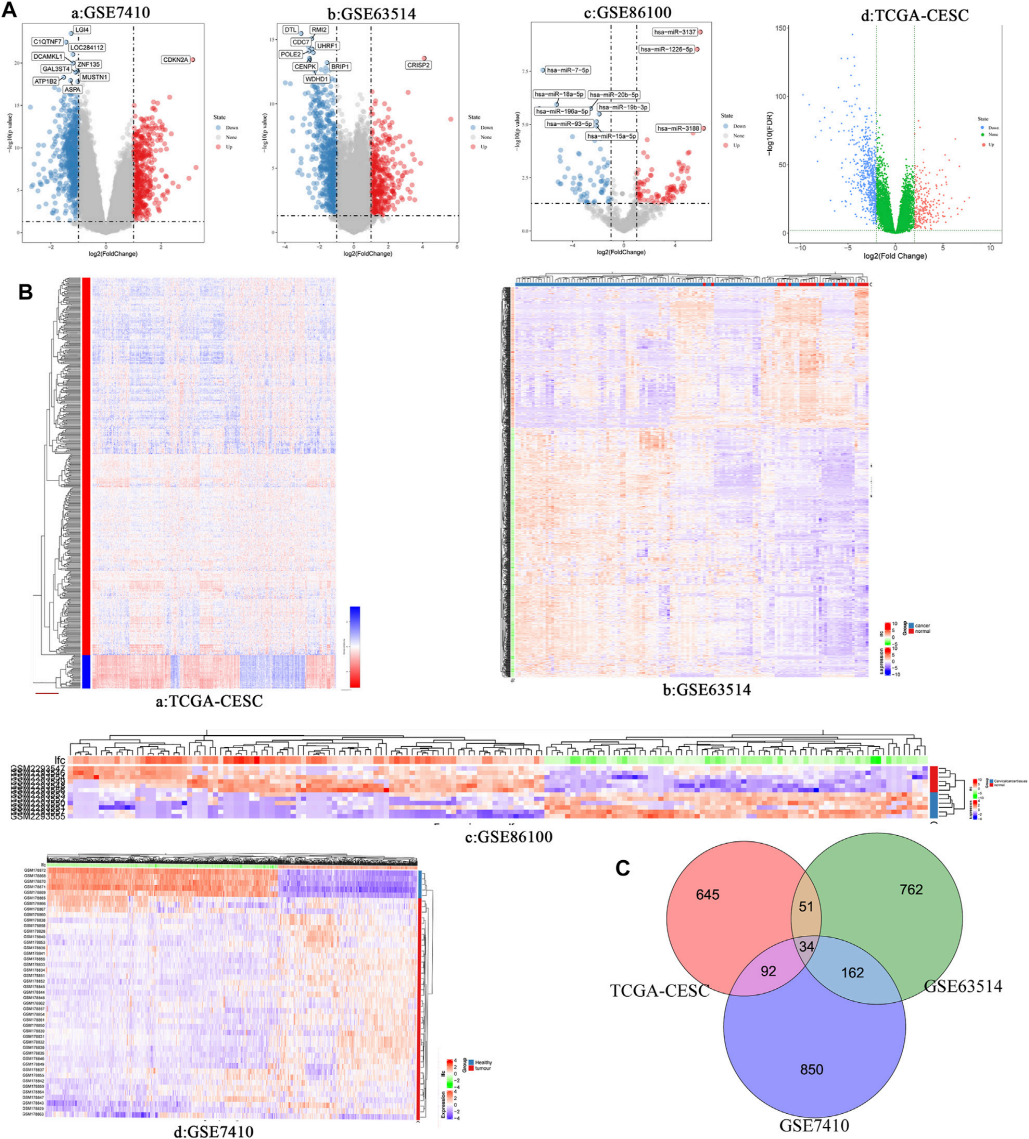
**(A)** Volcano plots of differentially expressed RNAs (mRNA and miRNA) in different data set of cervical cancer. a: GSE7410; b: GSE63514; c: GSE86100; d: TCGA-CESC. **(B)** Heatmap of differentially expressed RNAs (miRNA and mRNA) in different data set of cervical cancer. a: TCGA-CESC; b: GSE63514; c: GSE86100; d: GSE7410. **(C)** Venn diagram show the intersection results of differentially expressed genes in three different data sets of cervical cancer.

Three cervical cancer expression profile datasets GSE7410, GSE63514 and GSE86100 were downloaded from the GEO database. Table 1 shows the details of the three GEO datasets. GSE7410 and GSE63514 screened for differentially expressed mRNAs between cervical cancer and normal tissues, and GSE86100 screened for differentially expressed miRNAs. Based on screening criteria, GSE7410 and GSE63514 obtained 1,138 mRNAs (709 down-regulated and 429 up-regulated) and 1,009 mRNAs (641 down-regulated and 368 up-regulated), respectively (Figure 1A, Figures 1B–D, Figures 1A,B, Figure 1B, Supplementary Material S2 and Supplementary Material S3). GSE86100 obtained 166 miRNAs (74 down-regulated and 92 up-regulated) (Figures 1A–C, Figures 1B,C and Supplementary Material S4). In addition, 822 mRNAs (567 down-regulated and 255 up-regulated) were obtained (Figures 1A–D, Figures 1A,B and Supplementary Material S5) from TCGA-CESC. Subsequently, Venn diagram was used to compare mRNAs screened by GSE7410, GSE63514 and TCGA-CESC, and 34 overlapping differentially expressed mRNAs were obtained, including 22 up-regulated genes and 12 down-regulated genes (Figure 1C, Supplementary Table S1).

### Functional Enrichment Analysis of Overlapping Genes

**FIGURE 2:**
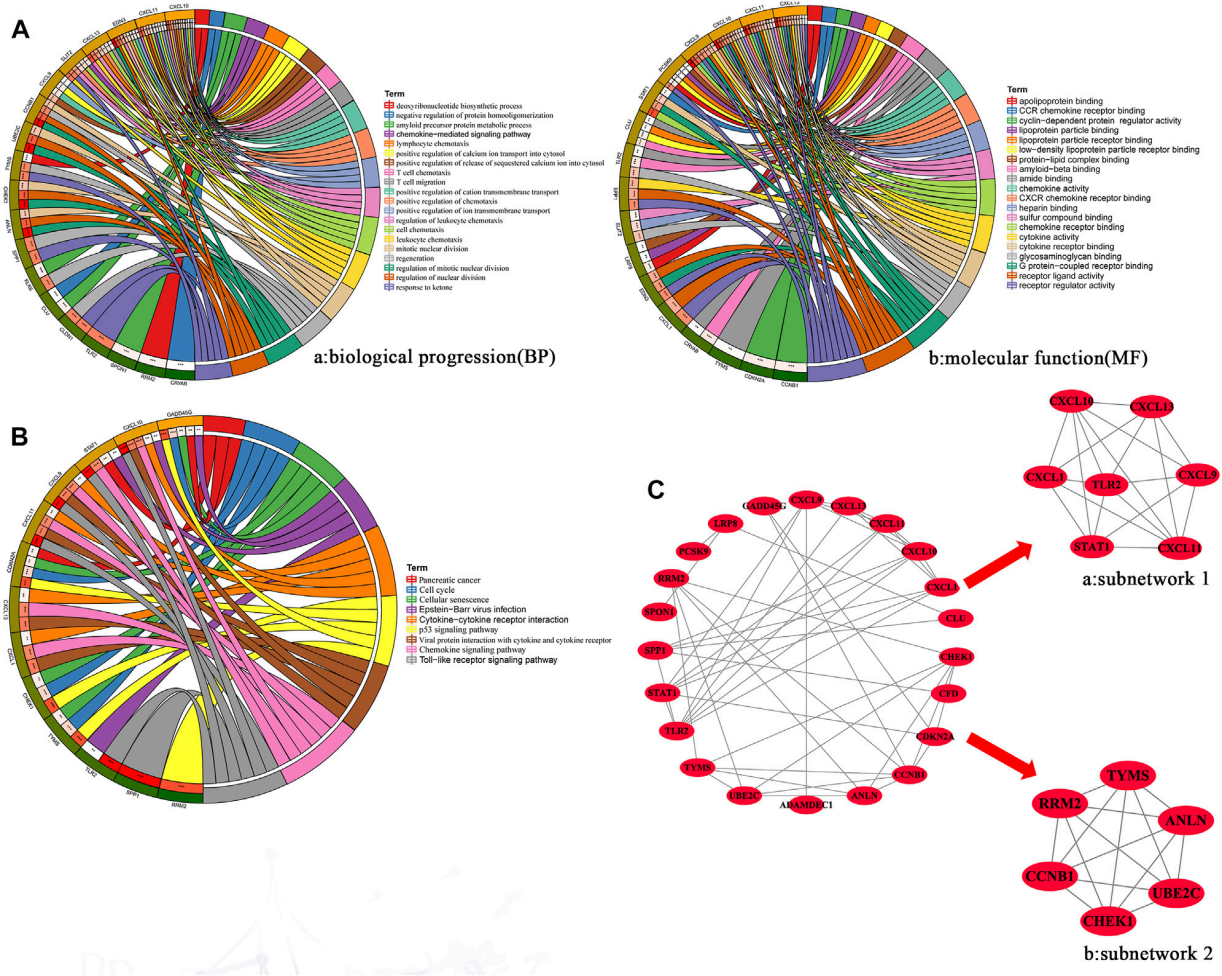
**(A)** GO enrichment analysis of hub overlapping genes were performed using DAVID online tool 6.8. MF: Molecular function: Biological processes. **(B)** KEGG enrichment analysis of hub overlapping genes were performed using DAVID online tool 6.8. **(C)** PPI network of the hub overlapping genes. The subnetwork 1(a) and subnetwork (b) of hub genes was screened by MCODE in cytoscape.

To explore the function and pathway of overlapping genes, we performed GO and KEGG pathway analyses. GO analysis showed that, in terms of biological progression, overlapping genes were mainly enriched in the regulation of mitotic nuclear division, T cell chemotaxis, regulation of nuclear division, leukocyte chemotaxis, positive regulation of the release of sequestered calcium into the cytosol, lymphocyte chemotaxis and mitotic nuclear division (Figure 2A). In terms of molecular function, overlapping genes were mainly enriched in CXCR chemokine receptor binding, chemokine receptor binding, chemokine activity, receptor regulator activity, G protein-coupled receptor binding, receptor ligand activity and cytokine activity (Figures 2A,B). Pathway enrichment analysis showed that overlapping genes interact with the Toll-like receptor signaling pathway, the p53 signaling pathway, viral protein interaction with cytokine and cytokine receptors, chemokine signaling pathways, the cell cycle and cellular senescence were related (Figure 2B).

### Construction and Analysis of PPI Networks for Overlapping Genes

We constructed PPI networks for 34 overlapping genes using the SRTING online database 11.5. The PPI network contains 22 nodes and 48 edges (Figure 2C). Then, two hub subnetworks were filtered through the MCODE plug-in from the PPI network (Figures 2A–C and Figures 2B,C). Subnetwork one contains the genes CXCL1, CXCL9, CXCL10, CXCL13, STAT1 and TLR2. Subnetwork two contains the genes ANLN, CCNB1, CHEK1, RRM2, TYMS and UBE2C. Based on the TCGA-CESC dataset, we mapped the expression profiles of 12 hub genes in cervical and normal tissues (Supplementary Figure S1). GO and KEGG analysis results showed that genes of subnetwork one were closely related to immunity, stress chemotaxis, angiogenesis, Toll-like receptor signaling pathway and chemokine signaling pathway (Supplementary Figure S2A). Genes in subnetwork two were mainly enriched in mitosis, the cell cycle and the p53 signaling pathway (Supplementary Figure S2B).

### Cox Regression and Proportional Risk Model Analysis of RRM2 and TYMS

**FIGURE 3:**
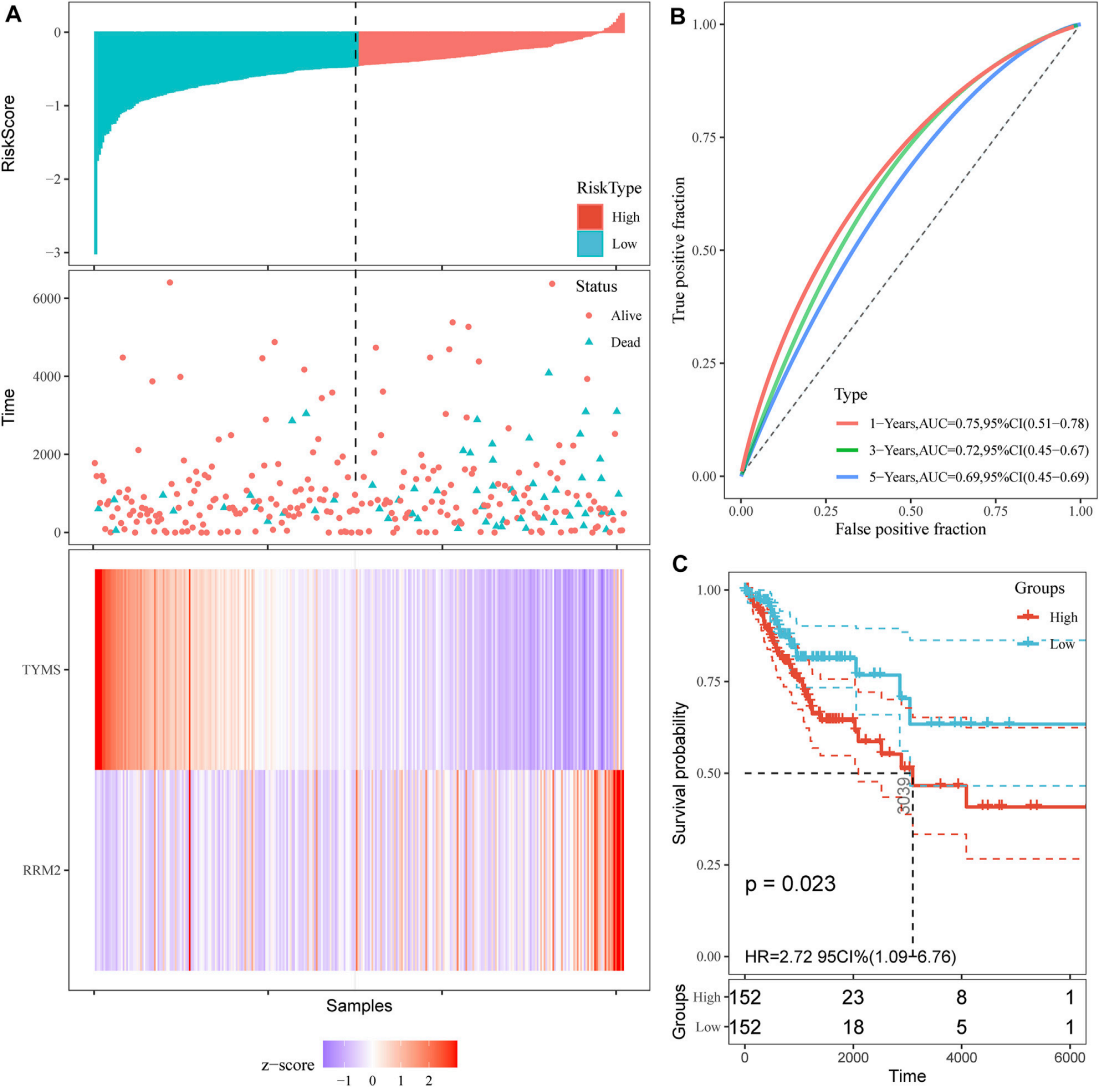
Prognostic risk score model analysis of TYMS and RRM2 in cervical cancer. **(A)** Risk score and cervical cancer patient of survival status distribution, and heatmap of TYMS and RRM2 expression by risk score. **(B)** ROC curves for predicting survival in cervical cancer patients by the risk score. **(C)** Kaplan–Meier curves for high and low-risk groups of cervical cancer patient by risk score.

First, overall survival (OS) data of cervical cancer patients were obtained from TCGA database. The survival curves of various subtypes and pathological parameters of cervical cancer were analyzed (Supplementary Figure S3). Subsequently, three genes were identified by univariate cox proportional risk regression model (*p* < 0.05) (Supplementary Figure S4, Table 2). Two further prognostic genes: RRM2 and TYMS, were selected by a multivariate Cox proportional risk regression model (Table 2). These two genes were also independent prognostic genes (*p* < 0.05). Risk prognosis model was established based on multivariate Cox screening results. The risk score was calculated as follows: mRNA risk score = (0.256)×expression (RRM2)+(-0.333)×expression (TYMS). Patients in TCGA-CESC were divided into high and low-risk groups based on the median risk score. The risk score of the TCGA-CESC dataset is shown in Figure 3. Figure 3A shows the details of the risk score. Kaplan-Meier survival analysis showed that the prognosis of high-risk group was worse than that of low-risk group (Figure 3C). The AUCs at 1 year, 3 and 5 years were 0.75, 0.72 and 0.69 respectively (Figure 3B). These results show that prognostic gene markers (RRM2 and TYMS) have good survival prediction ability, indicating that the model can effectively predict the prognosis of cervical cancer patients.

**TABLE 2:** Prognostic value of 12 hub genes in the cervical cancer patients of the TCGA cohort.

Hub genes	Univariate cox analysis		Multivariate cox analysis		Coefficient
	Hazard ratio (95% CI)	*p*-value	Hazard ratio (95% CI)	*p*-value	
CXCL1	2.29 (1.40–3.76)	0.033^*^	1.13 (1.00–1.27)	0.174	0.1181
CXCL9	0.42 (0.22–0.80)	0.077	0.91 (0.83–1.01)	0.071	-0.0908
CXCL10	0.63 (0.39–1.01)	0.160	0.95 (0.86–1.04)	0.250	-0.0560
CXCL13	0.55 (0.35–0.88)	0.360	0.94 (0.86–1.02)	0.129	-0.0667
STAT1	0.65 (0.40–1.03)	0.330	0.90 (0.7–1.13)	0.370	-0.1043
TLR2	0.58 (0.35–0.96)	0.180	0.85 (0.69–1.06)	0.160	-0.1578
ANLN	1.77 (1.10–2.83)	0.087	1.46 (1.03–2.07)	0.341	0.3790
CCNB1	0.59 (0.37–0.96)	0.320	0.87 (0.57–1.31)	0.493	-0.1450
CHEK1	0.80 (0.49–1.30)	0.910	1.08 (0.71–1.65)	0.715	0.0784
RRM2	1.40 (0.87–2.26)	0.029^*^	1.22 (0.81–1.83)	0.034^*^	0.2560
TYMS	0.47 (0.29–0.77)	0.041^*^	0.76 (0.52–1.12)	0.048^*^	-0.3332
UBE2C	0.75 (0.45–1.26)	0.510	1.00 (0.67–1.49)	0.997	8.00E-04

“*” represents *p* < 0.05.

### Co-Expression Network Construction

**FIGURE 4:**
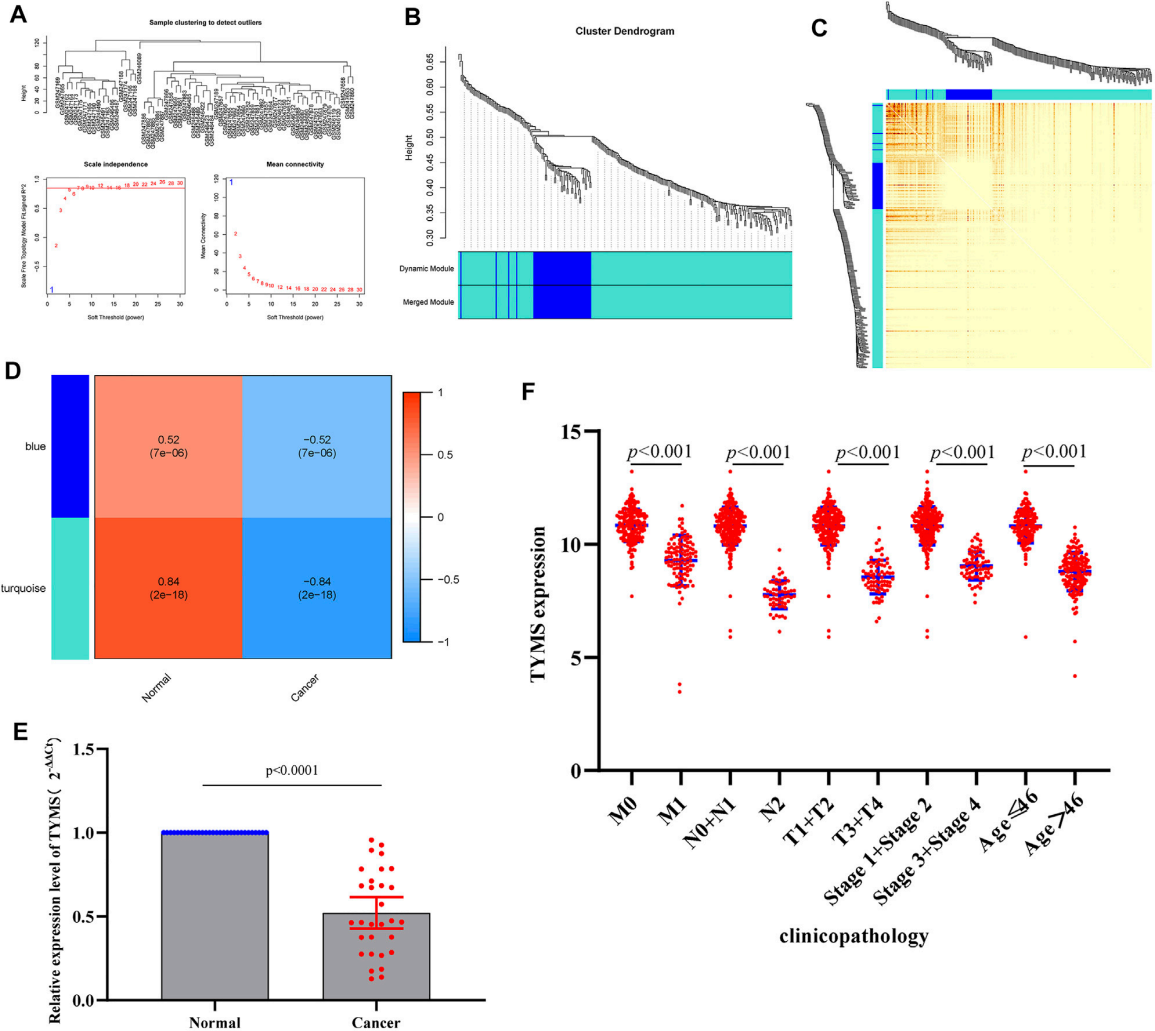
**(A)** a scale-free network of samples in GSE9750 data. **(B)** Module Dendrogram of genes in GSE9750 data. **(C)** Heatmap of the topological overlap map. **(D)** Module−trait relationships (normal sample vs cervical cancer sample). **(E)** RT-qPCR results of the expression levels of TYMS in 30 paired cervical cancer tissues and their corresponding adjacent normal tissues. The data were shown as mean ± standard deviations. **(F)** Correlation analysis of the expression of TYMS with clinicopathological parameters.

The coexpression network was constructed by (WGCNA) to further identify genes strongly associated with cervical cancer. First, the optimal soft threshold *β* = 1 was obtained through analysis, and the scale-free network was constructed (Figure 4A). Finally, two gene modules were obtained for further analysis (Figure 4B). From the heatmap of the topological overlap map, it can be seen that there was a high correlation between the genes in the module (Figure 4C). As shown in Figure 4D, the turquoise module was identified as the most specific module with a correlation coefficient of 0.84 (p = 2E-18). Therefore, this module was selected as clinically important for further analysis. By comparing the hub genes screened by multivariate Cox analysis with the genes in the turquoise module, we obtained a common key gene TYMS (Supplementary Table S2). This suggests that TYMS may play a very important role in the progression of cervical cancer.

### Low Expression of TYMS is Associated With Clinical Progression in Patients With Cervical Cancer

To investigate the expression of TYMS in cervical cancer, qRT-PCR was used to detect the expression levels of TYMS in cervical cancer tissue samples and adjacent tissues. The results showed that TYMS was significantly down-regulated in cervical cancer (Figure 4E), which was consistent with GEO database analysis results. Subsequently, we downloaded the expression data of TYMS and the clinical characteristics of cervical cancer patients from TCGA-CESC, and evaluated the relationship between the expression level of TYMS and various clinicopathological parameters. The analysis showed that down-regulation of TYMS expression was significantly correlated with histological grading (*p* < 0.001), clinical stage (*p* < 0.001) and patient age (*p* < 0.001) (Figure 4F). These results suggest that cervical cancer patients with low expression of TYMS are more likely to have more advanced tumor status, grade and stage than those with high expression of TYMS.

### GSEA of TYMS and its Expression in Relation to Immune Neoantigens, Tumor Mutation Load, Microsatellite Instability and Methylase Expression

**FIGURE 5:**
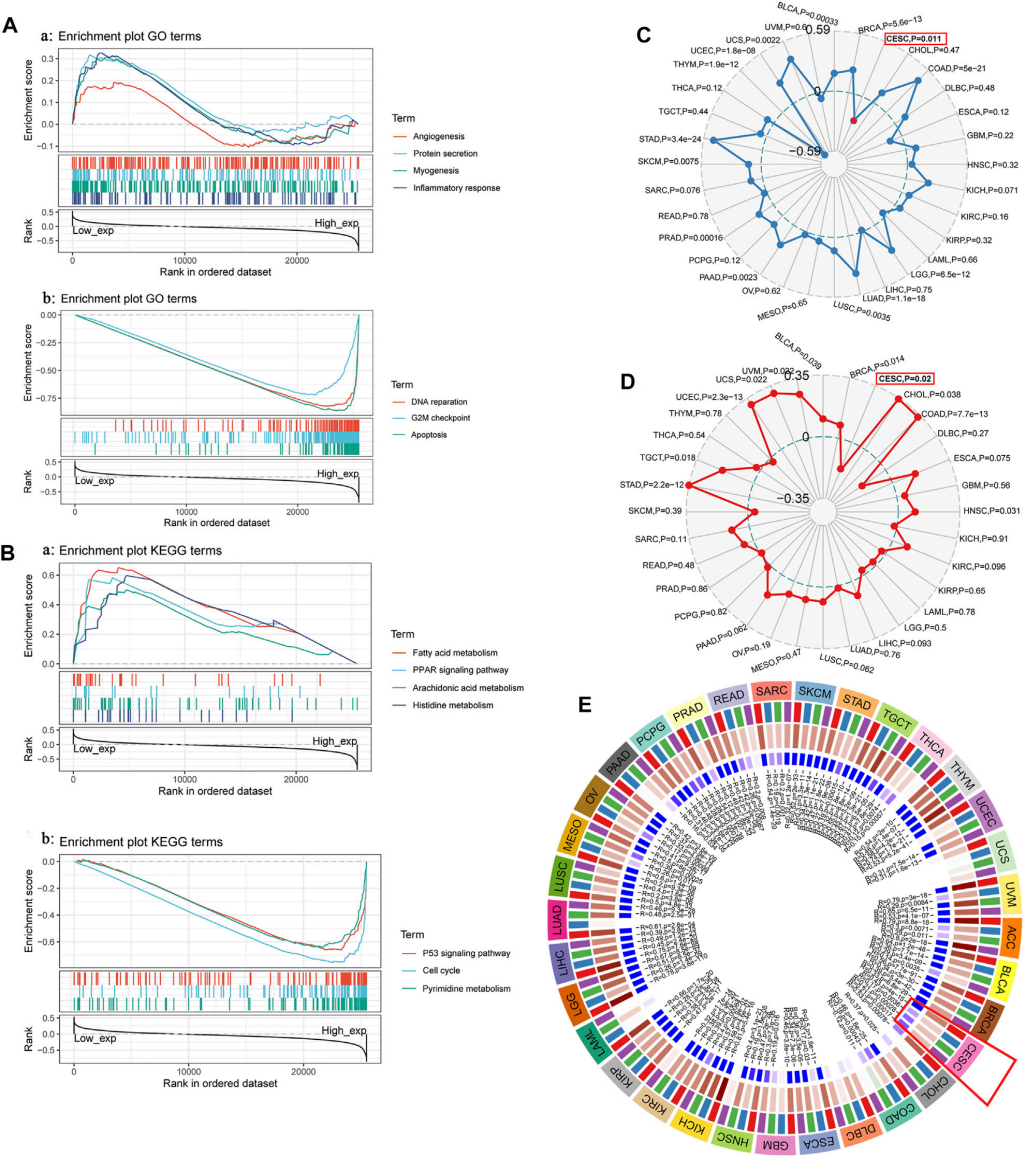
**(A)** and **(B)** GSEA investigation of TYMS. **(C)** The radar diagram shown the correlation between TYMS gene expression and tumor mutational burden (TMB). The red dots represent the correlation between TYMA and TMB in cervical cancer. The dotted blue circle in the middle represents the dividing line between positive and negative correlations. **(D)**The radar diagram shown the correlation between TYMS gene expression and microsatellite instability (MSI). The red box represents the association between TYMS and MSI in cervical cancer. The dotted circle in the middle represents the dividing line between positive and negative correlations. **(E)** Correlation between TYMS gene expression and four methyltransferases (DNMT1: red, DNMT2: blue, DNMT3A: green, DNMT3B: purple) expression. The red box represents the association between TYMS and methyltransferases in cervical cancer.

To further analyze and explore the potential biological functions of TYMS, we performed GSEA on TYMS. As shown in Figure 5A, angiogenesis, myogenesis, inflammatory response and protein levels were significantly positively correlated with TYMS expression at GO concentrations. DNA repair, G2M checkpoint and apoptosis were negatively correlated with TYMS expression (Figures 5A,B). KEGG pathway analysis showed that the pathways with a significant positive correlation with TYMS expression included fatty acid metabolism, the PPAR signaling pathway, fatty acid metabolism, arachidonic acid metabolism and histidine metabolism. The p53 signaling pathway, cell cycle and pyrimidine metabolism were significantly negatively correlated with TYMS expression (Figures 5A,B and Figure 5B). These results suggest that essential cell cycle control, amino acid metabolic pathways and regeneration processes are closely related to TYMS expression in patients with cervical cancer. In addition, we also analyzed the relationships between TYMS expression and immune neoantigens, tumor mutational burden, microsatellite instability and methylase expression. The results showed that the expression of TYMS was negatively correlated with tumor mutational burden (Figure 5C) and microsatellite instability (Figure 5D), and positively correlated with the expression of four methyltransferases (Figure 5E, DNMT1: red, DNMT2: blue, Dnmt3A: green and DNMT3B: purple). These results also show that low-level TYMS may easily leads to cell mutation, resulting in tumor formation or deterioration.

### Interference With TYMS Expression can Regulate HeLa cell Proliferation, Migration, Apoptosis and Invasion

**FIGURE 6:**
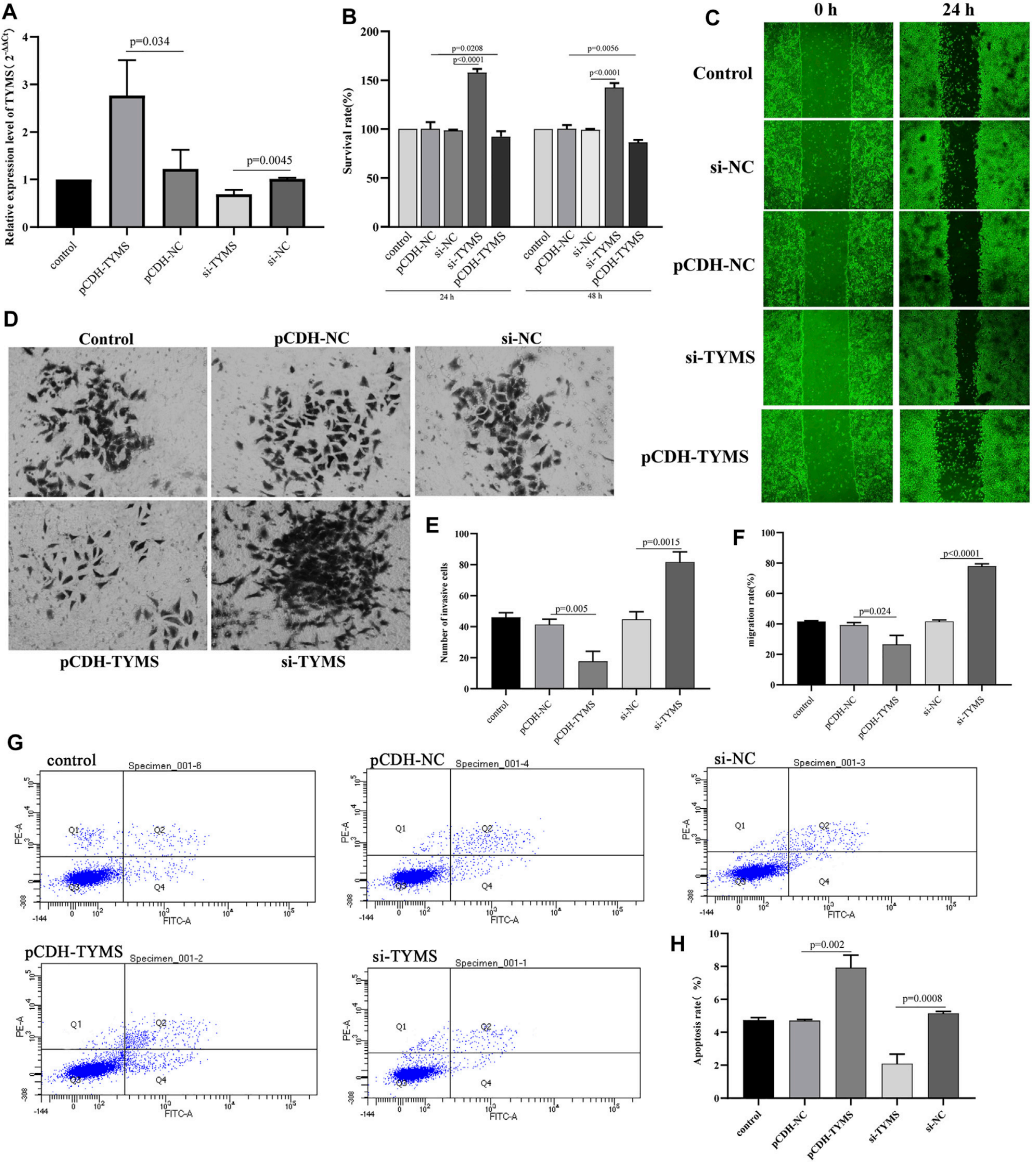
**(A)** QPCR results of the transfection efficiency in HeLa cell with TYMS overexpressed plasmid and si-TYMS. **(B)** The effects of si-TYMS and TYMS overexpressed on the proliferation activity of HeLa cells measured using the CCK-8. **(C)** and **(F)** The effects of si-TYMS and TYMS overexpressed on the migration of HeLa cells measured using wound-healing assay. **(D)** and **(E)** The effects of si-TYMS and TYMS overexpressed on the invasion of HeLa cells measured using *trans*-well assay. **(G)** and **(H)** The effects of si-TYMS and TYMS overexpressed on the apoptosis of HeLa cells measured using flow cytometry.

To further verify the analysis results of the biological function of TYMS, gain-of-function analysis was performed on HeLa cells transfected with the TYMS vector. QRT-PCR results showed that TYMS was effectively expressed in HeLa cells after transfection (Figure 6A). Then, the cell proliferation was detected by CCK-8 assay. The results showed that compared with the control group, HeLa cells transfected with si-TYMS showed significant growth promotion. In contrast, TYMS overexpression showed the opposite effect (Figure 6B). The results of wound healing and transwell assay showed that silencing TYMS promoted cell migration and invasion, and overexpression of TYMS inhibited cell migration and invasion (Figure 6C, Figure 6D, Figure 6E and Figure 6F). However, flow cytometry showed that overexpression of TYMS significantly promoted the amount of apoptosis in HeLa cells, while the apoptosis rate was relatively low when TYMS was knocked out (Figure 6G and Figure 6H). These results suggest that TYMS may be involved in the development of cervical cancer.

### TYMS is a Direct Target Gene of miR-197-3p in Cervical Cancer

**FIGURE 7:**
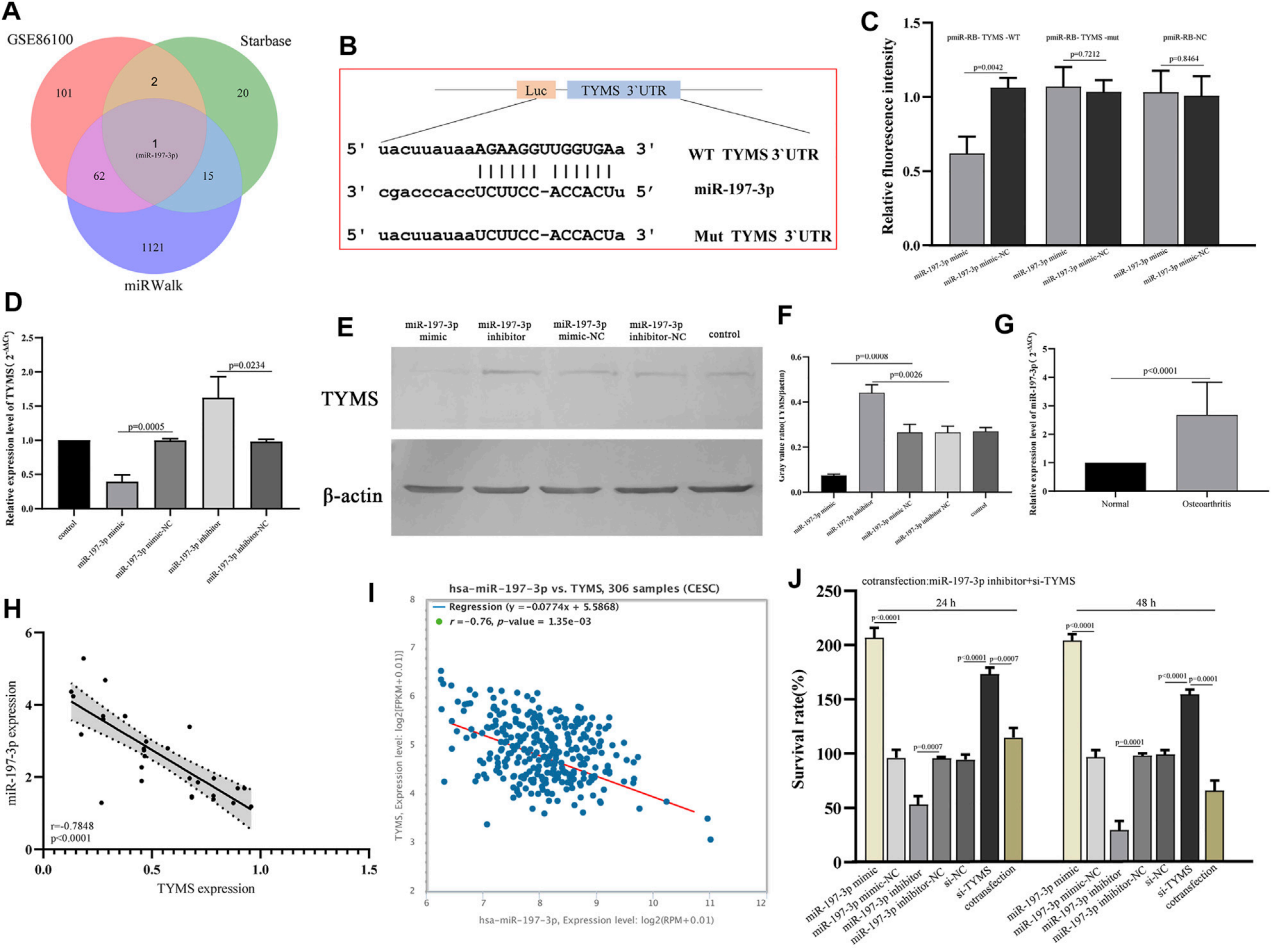
**(A)** Venn diagram show the intersection results of miRNAs from starbase, miRwalk and GSE86100. **(B)** The 3′UTR binding site sequence of miR-197-3p and TYMS. **(C)** Results of dual luciferase assay for miR-197-3p and TYMS. **(D)** QPCR results of expression changes of TYMS in HeLa cells after transfection with miR-197-3p mimic and inhibitor. **(E)** and **(F)** Western blot of expression changes of TYMS in HeLa cells after transfection with miR-197-3p mimic and inhibitor. **(G)** RT-qPCR results of the expression levels of miR-197-3p in 30 paired cervical cancer tissues and their corresponding adjacent normal tissues. The data were shown as mean ± standard deviations. **(H)** The correlation between the expression of TYMS and the expression of miR-197-3p in cervical cancer tissues and their corresponding adjacent normal tissues. **(I)** The correlation between the expression of TYMS and the expression of miR-197-3p in cervical cancer by starbase. **(J)** The effects of si-TYMS, pCDH-TYMS, miR-197-3p mimic, miR-197-3p inhibitor and co-transfection (si-TYMS and miR-197-3p inhibitor) on the proliferation activity of HeLa cells measured using the CCK-8.

To determine the potential mechanism of TYMS in cervical cancer, we analyzed the potential miRNAs targeted by TYMS using miRWalk and starbase online tools (Supplementary Material S6 and Supplementary Material S7). The analysis results were compared with the screened differentially expressed miRNAs, and a hub miRNA miR-197-3p was obtained (Figure 7A). The 3'UTR binding site sequences of miR-197-3p and TYMS are shown in Figure 7B. To verify the correlation between miR-197-3p and TYMS, luciferase reporter gene detection was performed. The results showed that miR-197-3p overexpression caused a significant decrease in luciferase activity in the wt-TYMS group. While, overexpression of miR-197-3p failed to inhibit luciferase activity in the mut-TYMS group (Figure 7C). In addition, qRT-PCR and western blot results showed that overexpression of miR-197-3p could reduce the expression of TYMS in HeLa cells. Similarly, the expression of TYMS was increased in HeLa cells after transfection with miR-197-3p inhibitor (Figure 7D, Figure 7E and Figure 7F). These results suggest a direct targeting relationship between TYMS and miR-197-3p.

### Overexpression of miR-197-3p Enhanced the Effect of TYMS on the Proliferation of HeLa Cells

To further confirm the regulatory relationship between miR-197-3p and TYMS, we detected the mRNA level of miR-197-3p in cervical cancer tissues. Detection results showed that miR-197-3p was up-regulated in cervical cancer tissues (Figure 7G). Then, correlation analysis was conducted on the expression levels of miR-197-3p and TYMS, and the results showed a significant negative correlation between miR-197-3p and TYMS (Figure 7H), which was consistent with the results of bioinformatics analysis (Figure 7I). The results of proliferation assay showed that overexpression of miR-197-3p significantly enhanced the proliferation activity of HeLa cells. In contrast, the miR-197-3p inhibitor inhibited HeLa cell proliferation (Figure 7J). In HeLa cells cotransfected with miR-197-3p inhibitor and si-TYMS, cell proliferation activity was significantly lower than that in the TYMS knockout group (Figure 7J). These results suggested that down-regulation of miR-197-3p could reverse the effect of the TYMS inhibitor on HeLa cell viability and suggest that miR-197-3p directly targets TYMS and negatively regulates its expression.

## Discussion

Worldwide, cervical cancer is the second most common malignancy in women and a leading cause of morbidity and mortality (Jedy-Agba et al.,2020; Shrestha et al.,2020). The greatest progress has been made in reducing cervical cancer deaths with the advent and implementation of screening programs (Silver and Kobri, 2020). Despite some advances in diagnostic accuracy, the prognosis for cervical cancer patients remains unsatisfactory. In previous studies, it has been found that mRNA can regulate the carcinogenesis of cervical cancer by binding miRNA (Ren et al., 2020). confirmed that miR-587 can promote the occurrence of cervical cancer by inhibiting interferon regulatory factor 6. MiR-125 inhibits the progression of cervical cancer by regulating VEGF and PI3K/AKT signaling pathways (Fu et al.,2020). In addition, Sun et al. (2020) proposed that miR-4429 sensitized cervical cancer cells to radiation by targeting RAD51. Similarly, our results suggest that TYMS is a protective factor against cervical cancer and is down-regulated in cervical cancer tissues. However, miR-197-3p directly targeted by TYMS is present a high level in cervical cancer tissues, and up-regulation of TYMS can inhibit the proliferation, invasion and migration of HeLa cells, and promote the apoptosis of HeLa cells. Up-regulation of miR-197-3p reversed the inhibitory effect of TYMS overexpression on HeLa cell proliferation. This evidence suggests that miR-197-3p/TYMS plays an important role in the progression of cervical cancer.

In this study, based on the expression profile data of cervical cancer in GEO and TCGA, through PPI, functional enrichment, Kaplan-Meier plotter analysis, Cox univariate and multivariate analysis, WGCNA and other bioinformatics analysis methods, we screened the factor, TYMS, which was significantly correlated with the prognosis of cervical cancer. TYMS is located on chromosome 18p and encodes thymine synthetase (TS), which is an essential enzyme involved in DNA replication and repair, and plays an important role in the biosynthesis of dTMP (Gallegos-Arreola et al.,2018; Fu et al.,2019). Its role in tumor cell proliferation has been reported. TYMS overexpression is associated with poor disease-specific survival and local recurrence-free survival of various solid tumors such as lung cancer (Ding et al.,2020), gastric cancer (Cao et al.,2017) and prostate cancer (Burdelski et al.,2015). TYMS is more frequently expressed in patients with clinically advanced prostate cancer than in patients with advanced prostate cancer (Burdelski et al.,2015). In colorectal cancer, knockdown of TYMS can inhibit tumor cell proliferation and promote cell apoptosis (Varghese et al.,2019). We also found that the expression level of TYMS was correlated with the tissue grade, tumor grade and age of cervical cancer patients, and TYMS with low expression was more likely to have advanced tumor state, grade and staging. GSEA further found that TYMS may be involved in cell cycle, apoptosis, amino acid metabolism and other biological processes in cervical cancer. The expression level of TYMS was negatively correlated with microsatellite instability (MSI) and Tumor mutation burden (TMB), and positively correlated with methylase expression in DNMT1, DNMT2, DNMT3A and DNMT3B. MSI and TMB are predictive biomarkers of immune checkpoint inhibitors (ICIs), reflecting the number of mutations in somatic cells (Salem et al.,2020). Cancer is a disease characterized by abnormal and uncontrolled cell growth mainly caused by gene mutations, and its mutation forms affect the steady-state development of a series of key cell functions (Martínez-Jiménez et al., 2019), which indicates that high MSI and high TMB may lead to tumorigenesis or affect tumor progression. In addition, the precise control of chromatin kinetics depends on the coordination of DNA methylation and covalent histone modification, and the enzymes and chromatin factors involved in these processes are also regulated by various RNA. The loss of control of these processes will directly lead to the occurrence of various pathological processes, such as tumors (Köhler F and Rodríguez-Paredes, 2020). The expression of TYMS is related to the content of methylase, which indicates that TYMS may be involved in the cellular genetic epigenetic regulation process, and its abnormal expression may lead to the process out of control. These analyses need more experimental data to verify. However, the study of TYMS in cervical cancer has not been reported, and its regulatory mechanism on the progression of cervical cancer is still unknown.

Through dual-luciferase reporter gene and functional experiments, it was clarified that TYMS may be the target gene of miR-197-3p in cervical cancer. Moreover, interference with the miR-197-3p mimic reduced the expression level of TYMS. After transfection with the miR-197-3p inhibitor, the expression levels of TYMS at the mRNA and protein levels were enhanced. *In vitro* functional experiments showed that inhibition of miR-197-3p could reverse the effects of TYMS overexpression on the proliferation activity of HeLa cells. These observations suggest that miR-197-3p can promote the progression of cervical cancer by targeting TYMS. MiR-197-3p has been reported to be involved in several human cancers (Ni et al., 2019; Huang et al., 2020). MiR-197-3p is significantly down-regulated in ovarian cancer, and overexpression can inhibit the growth of ovarian cancer cells (Xie et al.,2020). MiRNAs generally exert their regulatory role by selectively targeting protein-coding genes. Similarly, miR-197-3p has been shown to target multiple genes in human cells, such as KLF10 (You et al.,2021), ABCA7(Xie et al.,2020) and EHD2 (Fan et al.,2020). However, TYMS as a target of miR-197-3p in any type of cancer cell has not been studied. In summary, miR-197-3p, as an upstream regulator of TYMS, affects the progression of cervical cancer through the posttranscriptional effects of TYMS, which is crucial for the tumorigenicity of cervical cancer. The results point toward the therapeutic implications of miR-197-3p in human cervical cancer cells. However, studies in different cell lines and *in vivo* conditions need further confirmation.

This study did not further evaluate the potential signaling pathway of miR-197-3p/TYMS in cervical cancer. These signaling pathways are responsible for tumor cell progression behavior. However, in the functional enrichment and GSEA data of this study, TYMS was mainly related to p53, the cell cycle and other signaling pathways. Therefore, future research will focus on the potential signaling pathway mechanism and the *in vivo* regulation of miR-197-3p/TYMS in cervical cancer.

## Conclusion

In conclusion, our data show that TYMS is an independent prognostic gene of cervical cancer, that affects the proliferation, migration, invasion and apoptosis of cervical cancer cells. Therefore, it has a potential role as a biomarker for cervical cancer. MiR-197-3p/TYMS is an effective pair of regulators of cervical cancer, and can provide a good data basis for the treatment of cervical cancer.

## Data Availability

The original contributions presented in the study are included in the article/Supplementary Material, further inquiries can be directed to the corresponding author.

## References

[B1] Barbieri I. Kouzarides T. (2020). Role of RNA Modifications in Cancer. Nat. Rev. Cancer 20 (6), 303–322. 10.1038/s41568-020-0253-2 32300195

[B2] Biewenga P. Buist M. R. Moerland P. D. van Themaat E. V. L. van Kampen A. H. C. ten Kate F. J. W. (2008). Gene Expression in Early Stage Cervical Cancer. Gynecol. Oncol. 108 (3), 520–526. 10.1016/j.ygyno.2007.11.024 18191186

[B3] Burdelski C. Strauss C. Tsourlakis M. C. Kluth M. Hube-Magg C. Melling N. (2015). Overexpression of Thymidylate Synthase (TYMS) Is Associated with Aggressive Tumor Features and Early PSA Recurrence in Prostate Cancer. Oncotarget 6 (10), 8377–8387. 10.18632/oncotarget.3107 25762627 PMC4480759

[B4] Cao Y. Zhang G. Wang P. Zhou J. Gan W. Song Y. (2017). Clinical Significance of UGT1A1 Polymorphism and Expression of ERCC1, BRCA1, TYMS, RRM1, TUBB3, STMN1 and TOP2A in Gastric Cancer. BMC Gastroenterol. 17 (1), 2. 10.1186/s12876-016-0561-x 28056823 PMC5217235

[B5] Chen W. Gao C. Liu Y. Wen Y. Hong X. Huang Z. (2008). Bioinformatics Analysis of Prognostic miRNA Signature and Potential Critical Genes in Colon Cancer. Front. Genet. 11, 478. 10.3389/fgene.2020.00478 PMC7296168 32582275

[B6] Clough E. Barrett T. (2016). The Gene Expression Omnibus Database. Methods Mol. Biol. 1418, 93–110. 10.1007/978-1-4939-3578-9_5 27008011 PMC4944384

[B7] den Boon J. A. Pyeon D. Wang S. S. Horswill M. Schiffman M. Sherman M. (2015). Molecular Transitions from Papillomavirus Infection to Cervical Precancer and Cancer: Role of Stromal Estrogen Receptor Signaling. Proc. Natl. Acad. Sci. USA 112 (25), E3255–E3264. 10.1073/pnas.1509322112 26056290 PMC4485108

[B8] Ding X. Gu Y. Jin M. Guo X. Xue S. Tan C. (2020). The Deubiquitinating Enzyme UCHL1 Promotes Resistance to Pemetrexed in Non-small Cell Lung Cancer by Upregulating Thymidylate Synthase. Theranostics 10 (13), 6048–6060. 10.7150/thno.42096 32483437 PMC7255002

[B9] Fan H. Liu T. Tian H. Zhang S. (2020). TUSC8 Inhibits the Development of Osteosarcoma by Sponging miR-197-3p and T-argeting EHD2. Int. J. Mol. Med. 46 (4), 1311–1320. 10.3892/ijmm.2020.4684 32945345 PMC7447318

[B10] Fu K. Zhang L. Liu R. Shi Q. Li X. Wang M. (2020). MiR-125 Inhibited Cervical Cancer Progression by Regulating VEGF and PI3K/AKT Signaling Pathway. World J. Surg. Onc 18 (1), 115. 10.1186/s12957-020-01881-0 PMC7261381 32473637

[B11] Fu Z. Jiao Y. Li Y. Ji B. Jia B. Liu B. (2019). TYMS Presents a Novel Biomarker for Diagnosis and Prognosis in Patients with Pancreatic Cancer. Medicine (Baltimore) 98 (51), e18487. 10.1097/MD.0000000000018487 31861032 PMC6940182

[B12] Gallegos-Arreola M. P. Zúñiga-González G. M. Sánchez-López J. Y. Naranjo-Cruz A. Y. Peralta-Leal V. Figuera L. E. (2018). TYMS 2R3R Polymorphism and DPYD [IVS]14+1G>A Mutation Genes in Mexican Colorectal Cancer Patients. Acta Biochim. Pol. 65 (2), 227–234. 10.18388/abp.2017_2338 29906295

[B13] Gao D. Zhang Y. Zhu M. Liu S. Wang X. (20162016). miRNA Expression Profiles of HPV-Infected Patients with Cervical Cancer in the Uyghur Population in China. PLoS One 11 (10), e0164701. 10.1371/journal.pone.0164701 PMC5072605 27764149

[B14] Goodall G. J. Wickramasinghe V. O. (2021). RNA in Cancer. Nat. Rev. Cancer 21 (1), 22–36. 10.1038/s41568-020-00306-0 33082563

[B15] Huang H. Chen Y.-F. Du X. Zhang C. (2020). Identification and Characterization of Tumorigenic Circular RNAs in Cervical Cancer. Cell Signal. 73, 109669. 10.1016/j.cellsig.2020.109669 32423867

[B16] Huang Q. Ma B. Su Y. Chan K. Qu H. Huang J. (2020). miR-197-3p Represses the Proliferation of Prostate Cancer by Regulating the VDAC1/AKT/β-catenin Signaling Axis. Int. J. Biol. Sci. 16 (8), 1417–1426. 10.7150/ijbs.42019 32210729 PMC7085225

[B17] Hutter C. Zenklusen J. C. (2018). The Cancer Genome Atlas: Creating Lasting Value beyond its Data. Cell 173 (2), 283–285. 10.1016/j.cell.2018.03.042 29625045

[B18] Jedy-Agba E. Joko W. Y. Liu B. Buziba N. G. Borok M. Korir A. (2020). Trends in Cervical Cancer Incidence in Sub-saharan Africa. Br. J. Cancer 123 (1), 148–154. 10.1038/s41416-020-0831-9 32336751 PMC7341858

[B19] Köhler F. Rodríguez-Paredes M. (2020). DNA Methylation in Epidermal Differentiation, Aging, and Cancer. J. Invest. Dermatol. 140 (1), 38–47. 10.1016/j.jid.2019.05.011 31427190

[B20] Linehan W. M. Ricketts C. J. (2019). The Cancer Genome Atlas of Renal Cell Carcinoma: Findings and Clinical Implications. Nat. Rev. Urol. 16 (9), 539–552. 10.1038/s41585-019-0211-5 31278395

[B21] Liu S. Wang W. Zhao Y. Liang K. Huang Y. (2020). Identification of Potential Key Genes for Pathogenesis and Prognosis in Prostate Cancer by Integrated Analysis of Gene Expression Profiles and the Cancer Genome Atlas. Front. Oncol. 10, 809. 10.3389/fonc.2020.00809 32547947 PMC7277826

[B22] Martínez-Jiménez F. Muiños F. Sentís I. Deu-Pons J. Reyes-Salazar I. Arnedo-Pac C. (2020). A Compendium of Mutational Cancer Driver Genes. Nat. Rev. Cancer 20 (10), 555–572. 10.1038/s41568-020-0290-x 32778778

[B23] Ni J. S. Zheng H. Huang Z. P. Hong Y. G. Ou Y. L. Tao Y. P. (2019). MicroRNA-197-3p A-cts as a P-rognostic M-arker and I-nhibits C-ell I-nvasion in H-epatocellular C-arcinoma. Oncol. Lett. 17 (2), 2317–2327. 10.3892/ol.2018.9848 30675297 PMC6341871

[B24] Rasmi R. R. Sakthivel K. M. Guruvayoorappan C. (2020). NF-κB Inhibitors in Treatment and Prevention of Lung Cancer. Biomed. Pharmacother. 130, 110569. 10.1016/j.biopha.2020.110569 32750649

[B25] Ren N. Liang B. Li Y. (2020). Identification of Prognosis-Related Genes in the Tumor Microenvironment of Stomach Adenocarcinoma by TCGA and GEO Datasets. Biosci. Rep. 40 (10), BSR20200980. 10.1042/BSR20200980 33015704 PMC7560520

[B26] Ren Y. Dong J. He P. Liang Y. Wu L. Wang J. (2020). miR‐587 Promotes Cervical Cancer by Repressing Interferon Regulatory Factor 6. J. Gene Med. 22 (11), e3257. 10.1002/jgm.3257 32749750

[B27] Salem M. E. Bodor J. N. Puccini A. Xiu J. Goldberg R. M. Grothey A. (2020). Relationship between MLH1 , PMS2 , MSH2 and MSH6 Gene‐specific Alterations and Tumor Mutational burden in 1057 Microsatellite Instability‐high Solid Tumors. Int. J. Cancer 147 (10), 2948–2956. 10.1002/ijc.33115 32449172 PMC7530095

[B28] Scotto L. Narayan G. Nandula S. V. Arias-Pulido H. Subramaniyam S. Schneider A. (2008). Identification of Copy Number Gain and Overexpressed Genes on Chromosome Arm 20q by an Integrative Genomic Approach in Cervical Cancer: Potential Role in Progression. Genes Chromosom. Cancer 47 (9), 755–765. 10.1002/gcc.20577 18506748

[B29] Sharma S. Deep A. Sharma A. K. (2020). Current Treatment for Cervical Cancer: An Update. Acamc 20 (15), 1768–1779. 10.2174/1871520620666200224093301 32091347

[B30] Shin H. J. Han J. M. Choi Y. S. Jung H. J. (2020). Pterostilbene Suppresses Both Cancer Cells and Cancer Stem-like Cells in Cervical Cancer with Superior Bioavailability to Resveratrol. Molecules 25 (1), 228. 10.3390/molecules25010228 PMC6982958 31935877

[B31] Shrestha G. Mulmi R. Phuyal P. Thakur R. K. Siwakoti B. (2020). Experiences of Cervical Cancer Survivors in Chitwan, Nepal: A Qualitative Study. PLoS One 15 (11), e0234834. 10.1371/journal.pone.0234834 33151965 PMC7644025

[B32] Silver M. I. Kobrin S. (2020). Exacerbating Disparities?: Cervical Cancer Screening and HPV Vaccination. Prev. Med. 130, 105902. 10.1016/j.ypmed.2019.105902 31730943

[B33] Stelzle D. Tanaka L. F. Lee K. K. Ibrahim Khalil A. Baussano I. Shah A. S. V. (2021). Estimates of the Global burden of Cervical Cancer Associated with HIV. Lancet Glob. Health 9 (2), e161–e169. 10.1016/s2214-109x(20)30459-9 33212031 PMC7815633

[B34] Sun H. Fan G. Deng C. Wu L. (2020). miR‐4429 Sensitized Cervical Cancer Cells to Irradiation by Targeting RAD51. J. Cel Physiol 235 (1), 185–193. 10.1002/jcp.28957 31190335

[B35] Varghese V. Magnani L. Harada-Shoji N. Mauri F. Szydlo R. M. Yao S. (2019). FOXM1 Modulates 5-FU Resistance in Colorectal Cancer through Regulating TYMS Expression. Sci. Rep. 9 (1), 1505. 10.1038/s41598-018-38017-0 30728402 PMC6365533

[B36] Wang R. Pan W. Jin L. Huang W. Li Y. Wu D. (2020). Human Papillomavirus Vaccine against Cervical Cancer: Opportunity and challenge. Cancer Lett. 471, 88–102. 10.1016/j.canlet.2019.11.039 31812696

[B37] Xie W. Shui C. Fang X. Peng Y. Qin L. (2020). miR-197-3p Reduces Epithelial-Mesenchymal Transition by Targeting ABCA7 in Ovarian Cancer Cells. 3 Biotech. 10 (8), 375. 10.1007/s13205-020-02362-7 PMC7403231 32832335

[B38] Xu Y. Wu G. Li J. Li J. Ruan N. Ma L. (2020). Screening and Identification of Key Biomarkers for Bladder Cancer: A Study Based on TCGA and GEO Data. Biomed. Res. Int. 2020, 1–20. 10.1155/2020/8283401 PMC7003274 32047816

[B39] Yang L. Zeng W. Sun H. Huang F. Yang C. Cai X. (2020). Bioinformatical Analysis of Gene Expression Omnibus Database Associates TAF7/CCNB1, TAF7/CCNA2, and GTF2E2/CDC20 Pathways with Glioblastoma Development and Prognosis. World Neurosurg. 138, e492–e514. 10.1016/j.wneu.2020.02.159 32147549

[B40] You M. Zhang L. Zhang X. Fu Y. Dong X. (2021). MicroRNA-197-3p Inhibits the Osteogenic Differentiation in Osteoporosis by Down-Regulating KLF 10. Cia 16, 107–117. 10.2147/CIA.S269171 PMC7810594 33469278

