# Supplementary figures and images for: Identification of Potential Hub Genes and miRNA-mRNA Pairs Related to the Progression and Prognosis of Cervical Cancer Through Integrated Bioinformatics Analysis
Source: Front Genet. 2021 Dec 22;12:775006. doi: 10.3389/fgene.2021.775006 (PMC8727538; doi:10.3389/fgene.2021.775006)

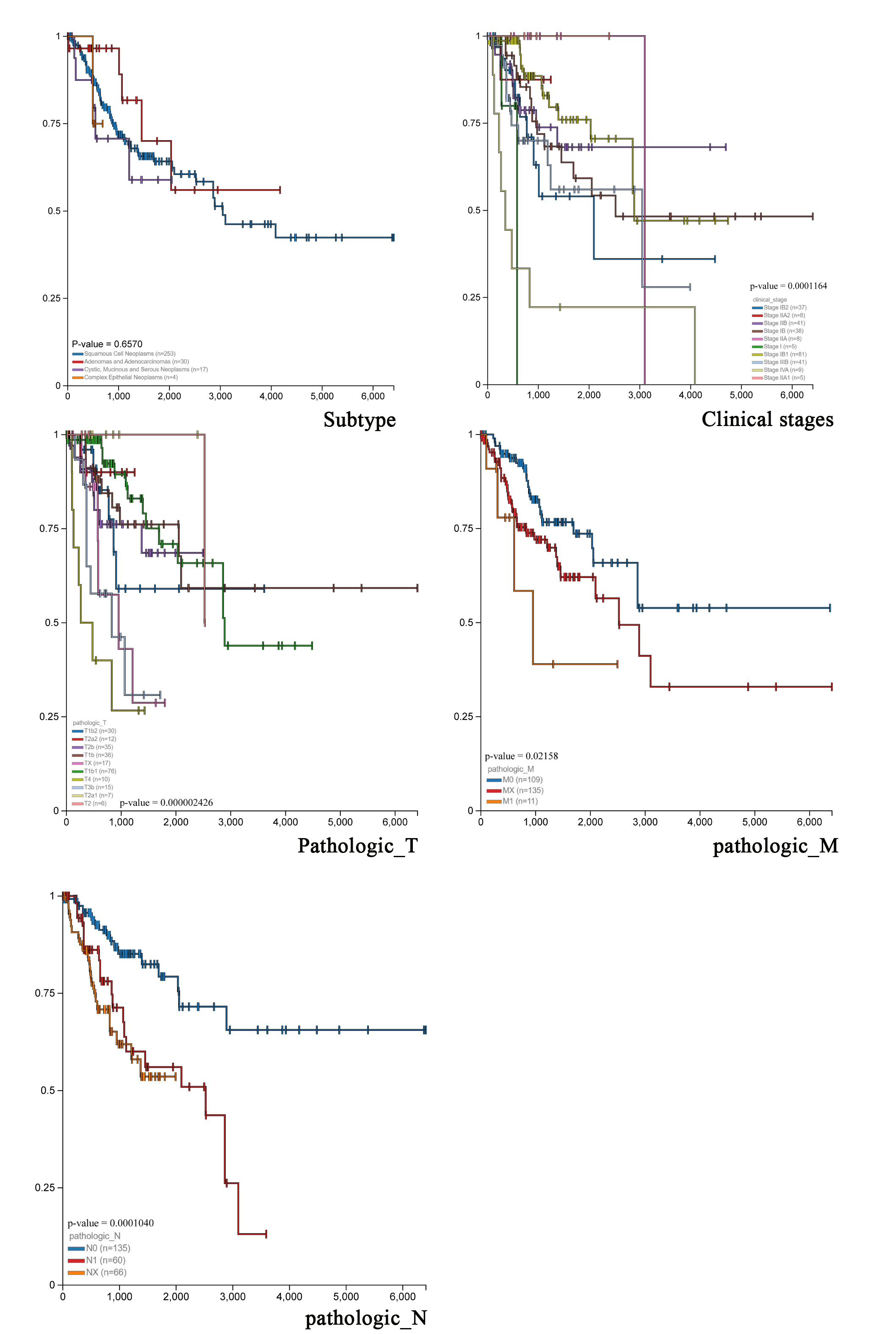

Supplement: Supplementary file 1 [file Image3.JPEG]

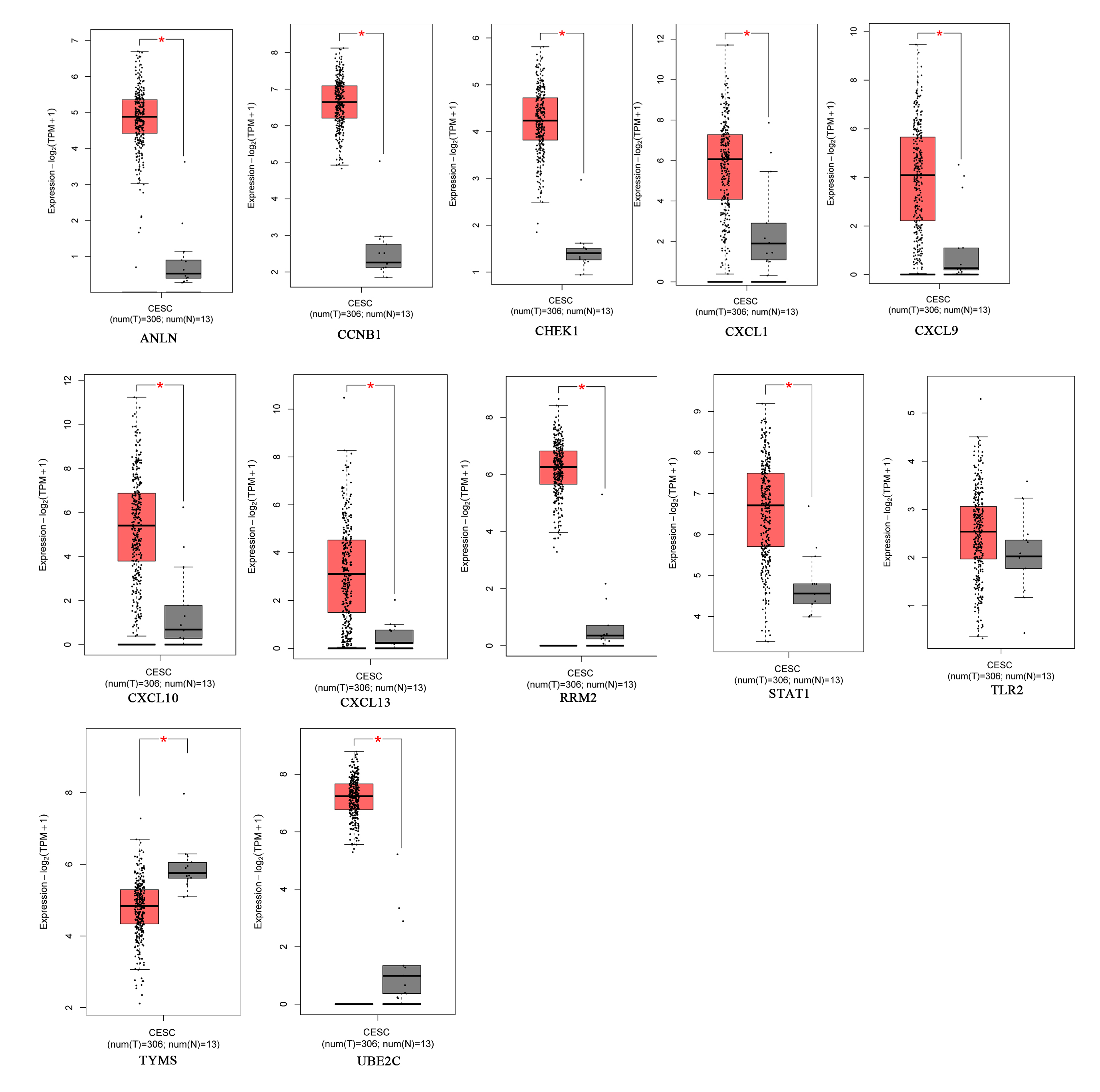

Supplement: Supplementary file 4 [file Image1.JPEG]

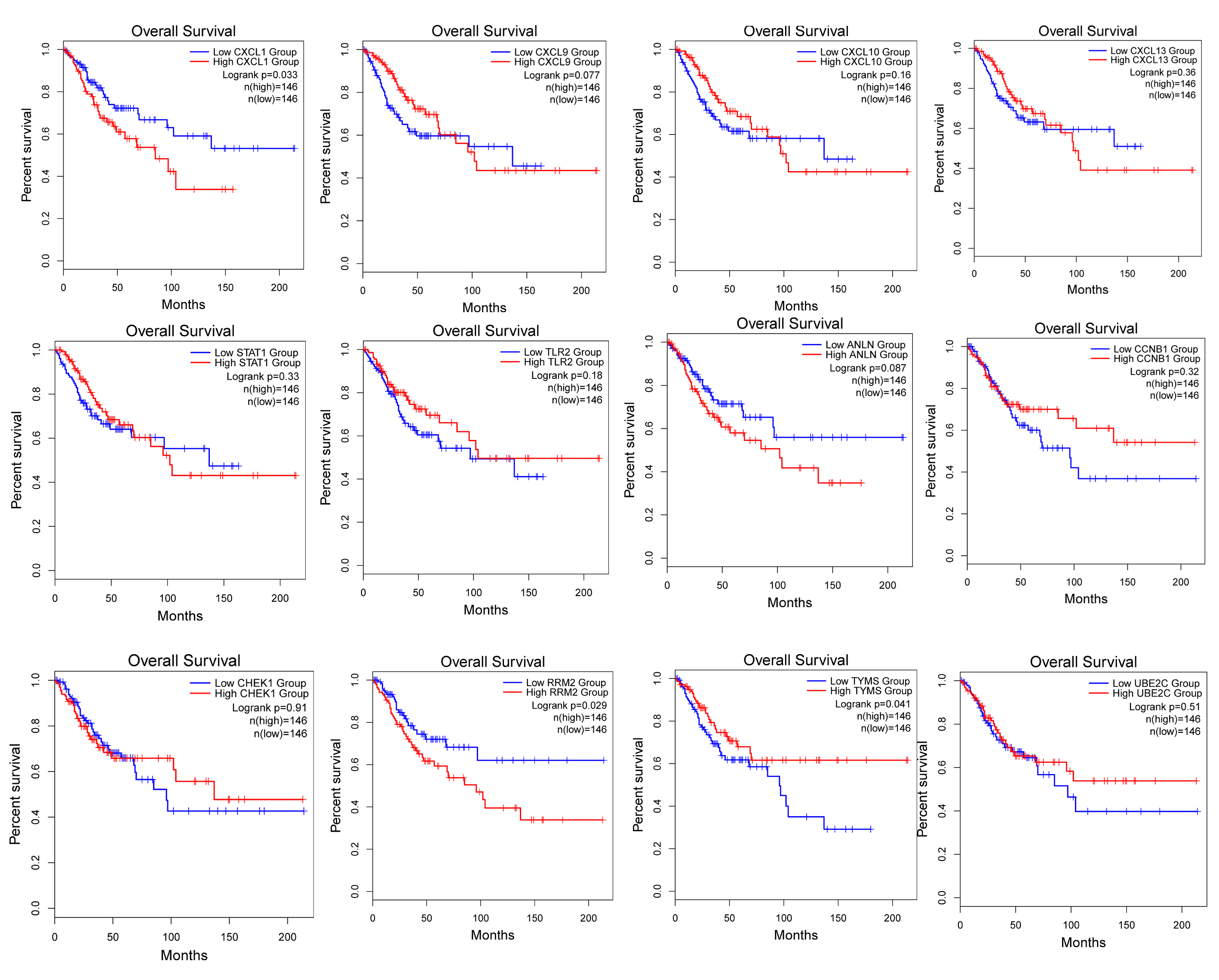

Supplement: Supplementary file 5 [file Image4.JPEG]

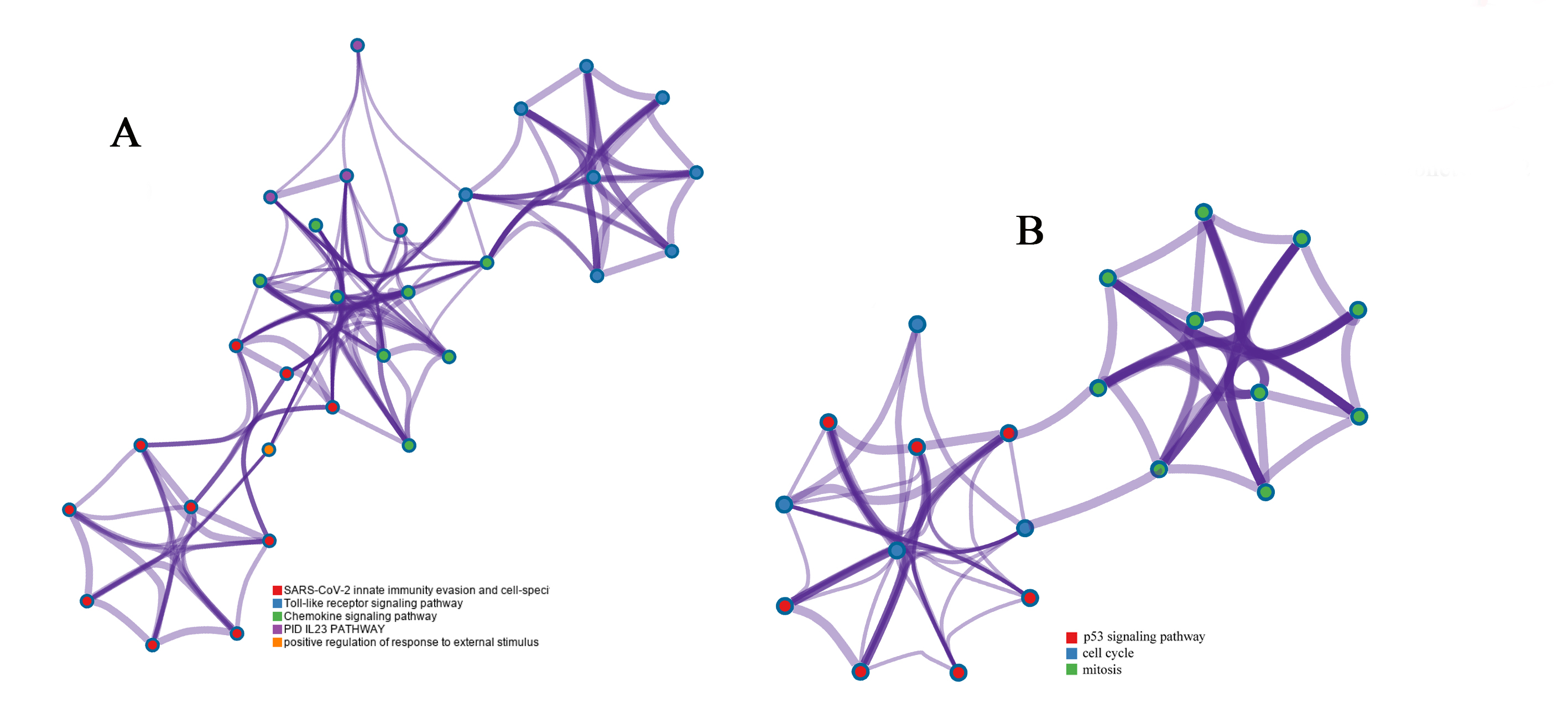

Supplement: Supplementary file 6 [file Image2.JPEG]
