# Supplementary material for: Identification of Potential Hub Genes and miRNA-mRNA Pairs Related to the Progression and Prognosis of Cervical Cancer Through Integrated Bioinformatics Analysis
Source: Front Genet. 2021 Dec 22;12:775006. doi: 10.3389/fgene.2021.775006 (PMC8727538; doi:10.3389/fgene.2021.775006)
Supplement: Supplementary file 2 [file Table1.DOCX]

Table 1 Information of GEO datasets.

| **Dataset** | **Platform** | | **Tumor** | **Normal** | **References** |
| --- | --- | --- | --- | --- | --- |
| GSE7410 | GPL1708 | Agilent-012391 Whole Human Genome Oligo Microarray G4112A | 40 | 5 | **Biewenga et al.,2008** |
| GSE63514 | GPL570 | Affymetrix Human Genome U133 Plus 2.0 Array | 28 | 24 | **Den *et al*.,2015** |
| GSE86100 | GPL19730 | Agilent-046064 Unrestricted Human miRNA V19.0 Microarray | 8 | 4 | **Gao *et al*.2016** |
| GSE9750 | GPL96 | Affymetrix Human Genome U133A Array | 43 | 23 | **Scotto *et al.*, 2008** |
