# Supplementary material for: Identification of Potential Hub Genes and miRNA-mRNA Pairs Related to the Progression and Prognosis of Cervical Cancer Through Integrated Bioinformatics Analysis
Source: Front Genet. 2021 Dec 22;12:775006. doi: 10.3389/fgene.2021.775006 (PMC8727538; doi:10.3389/fgene.2021.775006)
Supplement: Supplementary file 8 [file Table2.DOCX]

Table 2 Prognostic value of 12 hub genes in the cervical cancer patients of the TCGA cohort.

| **Hub genes** | **Univariate cox analysis** | | **Multivariate cox analysis** | | **Coefficient** |
| --- | --- | --- | --- | --- | --- |
|  | **Hazard ratio (95% CI)** | ***p*-value** | **Hazard ratio (95% CI)** | ***p*-value** |  |
| CXCL1 | 2.29(1.40-3.76) | 0.033^*^ | 1.13(1.00-1.27) | 0.174 | 0.1181 |
| CXCL9 | 0.42(0.22-0.80) | 0.077 | 0.91(0.83-1.01) | 0.071 | -0.0908 |
| CXCL10 | 0.63(0.39-1.01) | 0.160 | 0.95(0.86-1.04) | 0.250 | -0.0560 |
| CXCL13 | 0.55(0.35-0.88) | 0.360 | 0.94(0.86-1.02) | 0.129 | -0.0667 |
| STAT1 | 0.65(0.40-1.03) | 0.330 | 0.90(0.7-1.13) | 0.370 | -0.1043 |
| TLR2 | 0.58(0.35-0.96) | 0.180 | 0.85(0.69-1.06) | 0.160 | -0.1578 |
| ANLN | 1.77(1.10-2.83) | 0.087 | 1.46(1.03-2.07) | 0.341 | 0.3790 |
| CCNB1 | 0.59(0.37-0.96) | 0.320 | 0.87(0.57-1.31) | 0.493 | -0.1450 |
| CHEK1 | 0.80(0.49-1.30) | 0.910 | 1.08(0.71-1.65) | 0.715 | 0.0784 |
| RRM2 | 1.40(0.87-2.26) | 0.029^*^ | 1.22(0.81-1.83) | 0.034^*^ | 0.2560 |
| TYMS | 0.47(0.29-0.77) | 0.041^*^ | 0.76(0.52-1.12) | 0.048^*^ | -0.3332 |
| UBE2C | 0.75(0.45-1.26) | 0.510 | 1.00(0.67-1.49) | 0.997 | 8.00E-04 |

“*” represents p<0.05.
