# Supplementary material for: Identification of Potential Hub Genes and miRNA-mRNA Pairs Related to the Progression and Prognosis of Cervical Cancer Through Integrated Bioinformatics Analysis
Source: Front Genet. 2021 Dec 22;12:775006. doi: 10.3389/fgene.2021.775006 (PMC8727538; doi:10.3389/fgene.2021.775006)
Supplement: Supplementary file 10 [file Table3.DOCX]

Supplementary table 1. Overlapping differentially expressed genes in adjacent non-tumor tissues and cervical cancer tissues from GSE7410, GSE63514 and TCGA-CESC.

|  | **Overlapping differentially expressed genes** |
| --- | --- |
| **up-regulated** | CFD, PPP1R3C, TYMS, EDN3, SLIT2, SLC16A9, NDN, SPON1, IGFBP6, CLU, CRYAB, KLK6, GADD45G |
| **down-regulated** | LRP8, CHEK1, CXCL13, TLR2, HS6ST2, SPP1, CLDN1, CXCL1, RRM2, ADAMDEC1, CCNB1, HIST1H1C, STAT1, CXCL11, CXCL10, S100P, ANLN, PCSK9, UBE2C, CXCL9, CDKN2A |
