# Supplementary material for: Identification of Potential Hub Genes and miRNA-mRNA Pairs Related to the Progression and Prognosis of Cervical Cancer Through Integrated Bioinformatics Analysis
Source: Front Genet. 2021 Dec 22;12:775006. doi: 10.3389/fgene.2021.775006 (PMC8727538; doi:10.3389/fgene.2021.775006)
Supplement: Supplementary file 11 [file Table4.DOCX]

Supplementary table 2 Hub genes screened by multivariate cox and the genes in turquoise module.

|  | **Genes** |
| --- | --- |
| Hub genes | RRM2, TYMS |
| Genes in turquoise module | ABLIM1，ACTB，ADAR，AHNAK，AIM1，AKR1B1，AKR1B10，AKR1C3，ALOX12，ALOX12B，ANXA1，ANXA2，APOD，AQP3，ARPC2，ASS1，ATF3，ATP5H，ATP5L，BASP1，BHLHE40，BST2，CALML3，CALML5，CAST，CCT5，CD24，CD9，CDH3，CEACAM6，CKS1B，CLCA2，CLCA4，CLIC3，CLTB，CRABP2，CRCT1，CRISP3，CRNN，CRYAB，CSTA，CSTB，CTSK，CXCL14，CXCL8，DBI，DCN，DDIT4，DNAJB1，DSC2，DSG1，DSG3，DSP，DSTN，DUSP1，DUT，DYNLL1，DYNLT3，ECM1，EGR1，EIF1，EMP1，ENDOU，FABP5，FAM162A，FGFBP1，FN1，FOS，FOSB，FTH1，FTH1P5，FTL，GABARAP，GJA1，GLTP，GPX3，GSTA4，GSTP1，H2AFZ，H3F3A，HDGF，HEBP2，HIGD1A，HLA-DRA，HMGN2，HOPX，HSP90AA1，HSPB1，HSPB8，HSPD1，IER2，IER3，IFI16，IFITM3，IGFBP3，IGHG1，Igk，IGK，IGLC1，IL1RN，ISG15，ITM2B，IVL，JTB，JUNB，JUP，KANK1，KLF4，KLF6，KLK10，KLK11，KLK12，KLK13，KLK7，KLK8，KRT1，KRT10，KRT13，KRT14，KRT15，KRT16，KRT18，KRT19，KRT2，KRT4，KRT5，KRT6A，KRT6B，KRT8，LAPTM4A，LAPTM4B，LCN2，LGALS3，LOR，LPAR6，LY6D，LY6G6C，LYPD3，MAFB，MAL，MALL，MCM2，MCM6，NBL1，NFKBIA，P4HB，PABPC1，PABPC3，PDZK1IP1，PERP，PGAM1，PI3，PIM1，PKP1，PLBD1，PLP2，PLS3，PPA1，PPL，PPP1R3C，PRSS3，PTGDS，PTMA，PTTG1，RAB11A，RAB25，RHCG，RHOA，RHOB，RND3，RPL27A，RPLP2，RPN2，RPS11，RPS27A，S100A10，S100A11，S100A12，S100A14，S100A7，S100A8，S100A9，S100P，SCEL，SDC1，SEPP1，SERPINB1，SERPINB2，SERPINB3，SERPINB4，SERPINB5，SET，SFN，SGK1，SLC7A5，SLPI，SLURP1，SPARCL1，SPINK5，SPRR1A，SPRR1B，SPRR2B，SPRR2C，SPRR3，TFRC，TGFBI，TGM1，TGM3，TIMM8B，TIMP1，TIPARP，TM4SF1，TMEM45A，TMEM59，TMPRSS11D，TMPRSS11E，TMSB10，TRIM29，TRMT5，TSPO，TUBA1A，TUBA1C，TUBA4A，TUBB，TUBB2A，TUBB4B，TYMS，UBB，UBC，UBE2C，UBE2S，UPK1A，VAMP8，VDAC2，YPEL5，YWHAZ，ZFP36，ZNF185 |
